# Safety profile of herbal medicines submitted for marketing authorization in Tanzania: a cross-sectional retrospective study

**DOI:** 10.1186/s40545-023-00661-x

**Published:** 2023-11-20

**Authors:** Alambo K. Mssusa, Lone Holst, Godeliver Kagashe, Sheila Maregesi

**Affiliations:** 1 Tanzania Medicines and Medical Devices Authority, EPI External Mabibo, P.O. Box 77150, Dar Es Salaam, Tanzania; 2 Department of Global Public Health and Primary Care, University of Bergen, Årstadveien 17, Block D, 5009 Bergen, Norway; 3 Muhimbili University of Health and Allied Sciences, School of Pharmacy, P.O. Box 65013, Dar Es Salaam, Tanzania

**Keywords:** Tanzania Medicines and Medical Devices Authority (TMDA), Safety, Pharmacovigilance, Marketing Authorization, Registration, Dossiers, Herbal medicines, Herbal drugs, Alternative and complimentary medicines

## Abstract

**Background:**

The popular use of herbal medicines necessitates national regulatory authorities to have efficient mechanisms for the control of these products including marketing authorization (MA) and safety follow-up. Herbal medicines like conventional medicines require assessment of efficacy, safety and quality information before MA can be granted. However, the complete proof of safety is mainly based on the history of the long-term traditional use. Herbal medicines can cause adverse reactions due to various factors and thus require clinical trials to ensure their safety. Herbal medicines treatment practices involve combinations of different plants to achieve the desired effect while multiple herbal components have been known to cause herbal–herbal toxicity and interactions due to variety of complex active ingredients in plants. Compliance with regulatory requirements on herbal medicines has been shown to be difficult for manufacturers since different countries have different regulatory requirements with wide variations which results in the MA of very few herbal medicines. Limited studies on dossiers of marketing authorization of herbal medicines have been performed in other countries, with no studies in African regulatory system settings. The aim of this study is to determine the type of safety documentation that is submitted on herbal medicines application dossiers to support MA in Tanzania.

**Methods:**

A cross-sectional retrospective study of herbal medicines dossiers submitted at the Tanzania Medicines and Medical Devices Authority from 2009 to 2020 was conducted.

**Results:**

As many as 75% of the herbal products applications were combination products made by more than one herbal substance or plant. Out of 84 dossiers subjected to analysis the majority did not provide evidence of preclinical (55%) and clinical safety data (68%). Evidence of safety data in humans was mostly from the literature (70%) and not manufacturers’ clinical studies. Quality parameters with safety implications were not included in 48% and 23% of the active herbal substance and finished product specifications, respectively.

**Conclusion:**

Analysis of the herbal medicine dossiers submitted showed major deficiencies of safety data to support MA. Manufactures need to provide evidence to support the safety of their products for evidence-based regulatory decisions and to avoid multiple reviews of the applications.

**Supplementary Information:**

The online version contains supplementary material available at 10.1186/s40545-023-00661-x.

## Introduction

Herbal, alternative and complementary medicines are used for the treatment of various ailments worldwide in both industrialized and developing countries [1]. Herbal medicines are used either as an alternative or in conjunction with conventional medicines. The World Health Organization defines herbal medicine as “A plant-derived material or preparation with therapeutic or other human benefits which contains either raw or processed ingredients from one or more plants. In some traditions, materials of inorganic or animal origin may also be present” [2]. The popular use of herbal medicines necessitates national regulatory authorities to have efficient mechanisms for the control of these products including marketing authorization (MA) and safety follow-up [3–5].

Proof of evidence of the safety, efficacy and quality of herbal medicines before they are granted MA is important to ensure the safety of consumers in the same way as for conventional medicines [6, 7]. In contrast to the regulation of conventional medicines, some regulatory authorities, such as those in European countries, China and Japan, do not require proof of efficacy and safety through clinical trials for some herbal medicines [8, 9]. Safety and efficacy information is therefore substantiated by manufacturers through literature sources based on the long-term traditional use of the product in communities or a well-established use. Long-term traditional use refers to herbal medicines that have been used historically for several decades [6]. Herbal medicinal products with well-established medicinal use are those with an acceptable level of safety and a recognizable level of efficacy, whose active ingredients have been used within the European Community for at least 10 years [10].

Long-term traditional use or lack of adequate documentation of adverse reactions (ARs), however, does not prevent a herbal product from being unsafe or toxic since there is a possibility of the occurrence of ARs from inherent toxicity, unknown doses, adulteration or interactions with conventional medicines or supplements [11, 12]. Serious ARs such as acute kidney injury, hypertension, and heart failure, for instance, have been shown to be caused by *Glycyrrhiza glabra* (Liquorice), a herbal medicine with a long traditional use globally [13]. Plants containing aristolochic acid have been shown to cause neuropathy in studies conducted in China [14].

Clinical trials for herbal medicines, therefore, need to be conducted in a systematic manner to ensure adequate collection of safety and efficacy information as evidence for MA. There are a limited number of herbal medicines whose safety and efficacy have been well studied systematically in randomized controlled clinical trials (RCTs) [15, 16]. To support this, the World Health Organization (WHO) has issued Guidelines for the evaluation of safety and efficacy of herbal medicines and Guidelines for methodologies for research and evaluation of herbal medicines to assist researchers, manufacturers and regulatory authorities in generating and assessing data for the MA of herbal medicines [2, 17]. Furthermore, as the safety of herbal medicines is linked to their quality due to possible contamination with, e.g., heavy metals, pesticide residues, aflatoxins and other mycotoxins, evidence of quality for the different formulations and dosage forms should be established to ensure safety [18].

Herbal medicine treatment practices involve combinations of different plants to achieve the desired effect and therefore most herbal preparations on the market are combinations of multiple herbal components. Despite the perceived benefits of the herbal combinations claimed in Chinese practice and some scientific studies, the multiple herbal components have been known to cause herbal–herbal interactions due to a variety of complex active ingredients in plants [19]. Nevertheless, these combinations are believed to be safe by consumers since they have been traditionally used for decades [20]. Furthermore, manufacturers in the modern era are producing herbal products with combinations of different plants/ingredients and for different indications from those traditionally used [21]. This makes it challenging for regulators to establish the benefit–risk profile as there is a lack of sufficient data to support the safety and efficacy of the herbal substances/ingredients in the new multicomponent herbal products [22–24].

Compliance with regulatory requirements on herbal medicines has been shown to be difficult for manufacturers since different countries have different regulatory requirements with wide variations, which results in the MA of very few herbal medicines [25]. Some manufacturers of herbal medicines have limited knowledge of regulatory requirements in different countries and therefore opt to sell the products as food supplements, while they have therapeutic claims and basically fall into the category of herbal medicines [26, 27]. A regulatory decision to grant MA for a product is mainly based on the type of evidence that is submitted by the applicants to make a favourable benefit–risk balance to support their product.

Procedures for MA of herbal medicines are the same as for conventional medicines in some countries whereby the International Council for Harmonization of Technical Requirements for Pharmaceuticals for Human Use (ICH) Common Technical Document (CTD) format is used for compilation of the preclinical, clinical and quality data in the dossiers for MA [28, 29]. However, for herbal medicines, the requirements of study data in some countries on sections of the CTD such as module 4 (preclinical studies) and module 5 (clinical studies) are exempted in some of the products with long-term traditional use or when an ingredient is described in recognized official herbal monographs [30, 31]. Therefore, it is practically challenging to harmonize MA requirements for herbal medicines as some of the scientific information is missing for some products and categorization is different for each country.

In Tanzania, legal provisions for the control of herbal medicines are outlined in the Tanzania Medicines and Medical Devices Act, Cap 219 of 2003 [32], which mandates the Tanzania Medicines and Medical Devices Authority (TMDA) to control the registration, importation, inspection and safety monitoring of herbal medicines. Provisions for the MA of herbal medicines are contained in Part IV of the Act and Part II of the Tanzania, Food, Drugs and Cosmetics (Registration of Medicinal Products) Regulations 2015 [32, 33].

To provide guidance to herbal medicine applicants and manufacturers on the data requirements for safety, efficacy and quality of herbal medicines, the Guidelines on Submission of Documentation for Marketing Authorization of Herbal Medicinal Products were first developed by the TMDA in 2004 and subsequently revised in 2017 and 2020 to introduce the CTD format and elaborate on some submission requirements [34].

Limited studies have been performed to quantitatively assess and analyse regulatory dossier submissions for MA of herbal medicines [35–37], with no studies conducted in African regulatory authority settings. The aim of this study is to determine the type of safety documentation that is submitted on herbal medicine application dossiers to support MA in Tanzania.

## Methods

A cross-sectional retrospective study of herbal medicine dossiers submitted to the TMDA between 2009 and 2020 was conducted in 2021. The first author selected the names and code numbers of all products categorized as herbal medicines from the TMDA register and retrieved the dossiers in paper form from the TMDA archive and in electronic form from the TMDA internal database. Dossiers received from 2009 to 2016 were in paper form and CD-ROM, and those received from 2017 to 2020 were in electronic form.

The TMDA guidelines used prior to the introduction of the CTD were Guidelines for Application for Registration of Herbal Medicines in Tanzania (2004) [38]. The dossier requirements in the guidelines included sections on generic requirements, a summary of product characteristics, quality requirements, safety data and efficacy data. Data from the summary of product characteristics, safety, quality and package insert sections were retrieved. A descriptive analysis of the data was conducted.

After the introduction of the CTD, the Guidelines on Submission of Documentation for Marketing Authorization of Herbal Medicinal Products 2017 and revision in 2020 were used [34]. Dossier sections included general information, module 1 (administrative and product information), module 2 (overview and summaries), module 3 (quality), module 4 (non-clinical study reports) and module 5 (clinical study reports) [34]. Data from the summary of product characteristics, module 2 (summaries), module 4 (non-clinical), module 5 (clinical) and package inserts sections were retrieved and analysed.

Variables were identified from dossier sections received from both periods and were included in a Microsoft Excel 2018 data sheet [39]. The following variables were used for analysis:

1. Product information: unique identification number, botanical names, active herbal ingredients, formulation dosage forms, indications, safety category.
2. Preclinical variables: preclinical safety, preclinical toxicity, preclinical toxicity animal species and Good Laboratory Practice (GLP).
3. Clinical variables: clinical safety, clinical data source, clinical interactions, clinical overdose and Good Clinical Practice (GCP) evidence.
4. Quality variables with safety implications: specifications and batch analysis results for herbal substance/ingredients and finished products; Good Agricultural and Collection Practice (GACP), monograph type, purity/contaminant tests, batch analysis, Good Manufacturing Practice (GMP). The terms “herbal substances” and “herbal ingredients” will be used interchangeably in this paper.
5. Safety information variables on package inserts/summary of product characteristics: side effects, warnings, precautions, contraindications, overdose and interactions.

The assessed data in this study were for those applications that were submitted in the first round of submissions before being queried and resubmitted. A product is granted MA after the applicant responds to the queries or comments to assessor’s satisfaction. A product status will be considered “Queried” until the applicant satisfactorily responds to the queries. The terminology “Registered” is used to indicate that a product has been granted MA. A product from the same manufacturer that had the same ingredients and strength except for the flavouring agent was considered as one product in the assessment.

Quantitative data analysis was performed using SPSS Statistics software for Windows version 28 [40] and Microsoft Excel software. Descriptive statistics were used whereby measures of central tendencies (mean, mode and range) were determined. Frequencies and proportions were used for the categorical variables and the percentages were rounded.

Hypothesis testing for differences in proportions between the number of dossiers received and approved before introduction of CTD and post CTD was performed using the Fisher’s exact test, with the assumption of a normal distribution. The level of significance was determined at a p value of less than 0.05.

## Results

### Herbal medicine dossiers applications

**Fig. 1:**
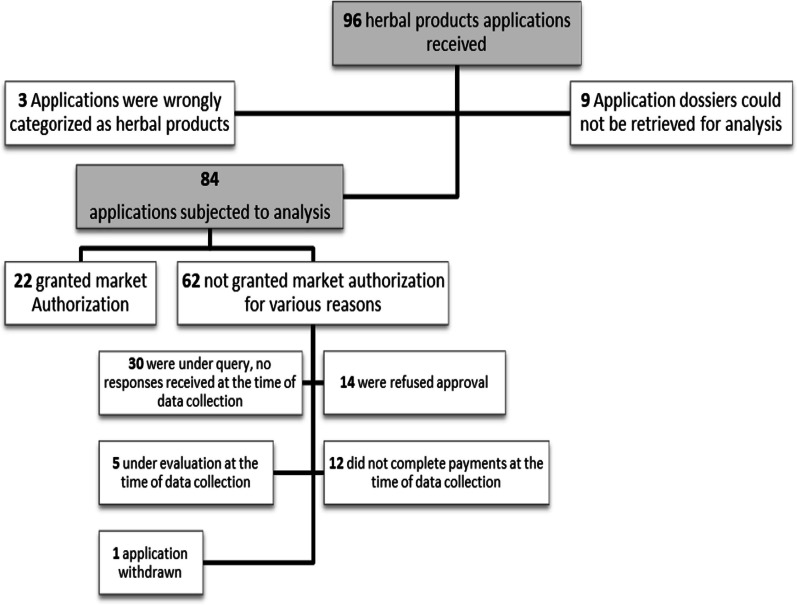
Flowchart of herbal product applications received at TMDA (2009–2020)

A total of 96 herbal medicine dossiers were submitted to the TMDA in paper form or online between 2009 and 2020. Out of the submitted dossiers, 84 could be retrieved and were subjected to analysis. Twenty-six percent of the herbal medicines were granted MA and 74% were not granted MA as depicted in Fig. 1.

The number of herbal medicine dossiers received by the TMDA varied from one to 15 per year, with an average of seven. The average number of herbal medicines registered was two per year, with a minimum of one and a maximum of five.

**Fig. 2:**
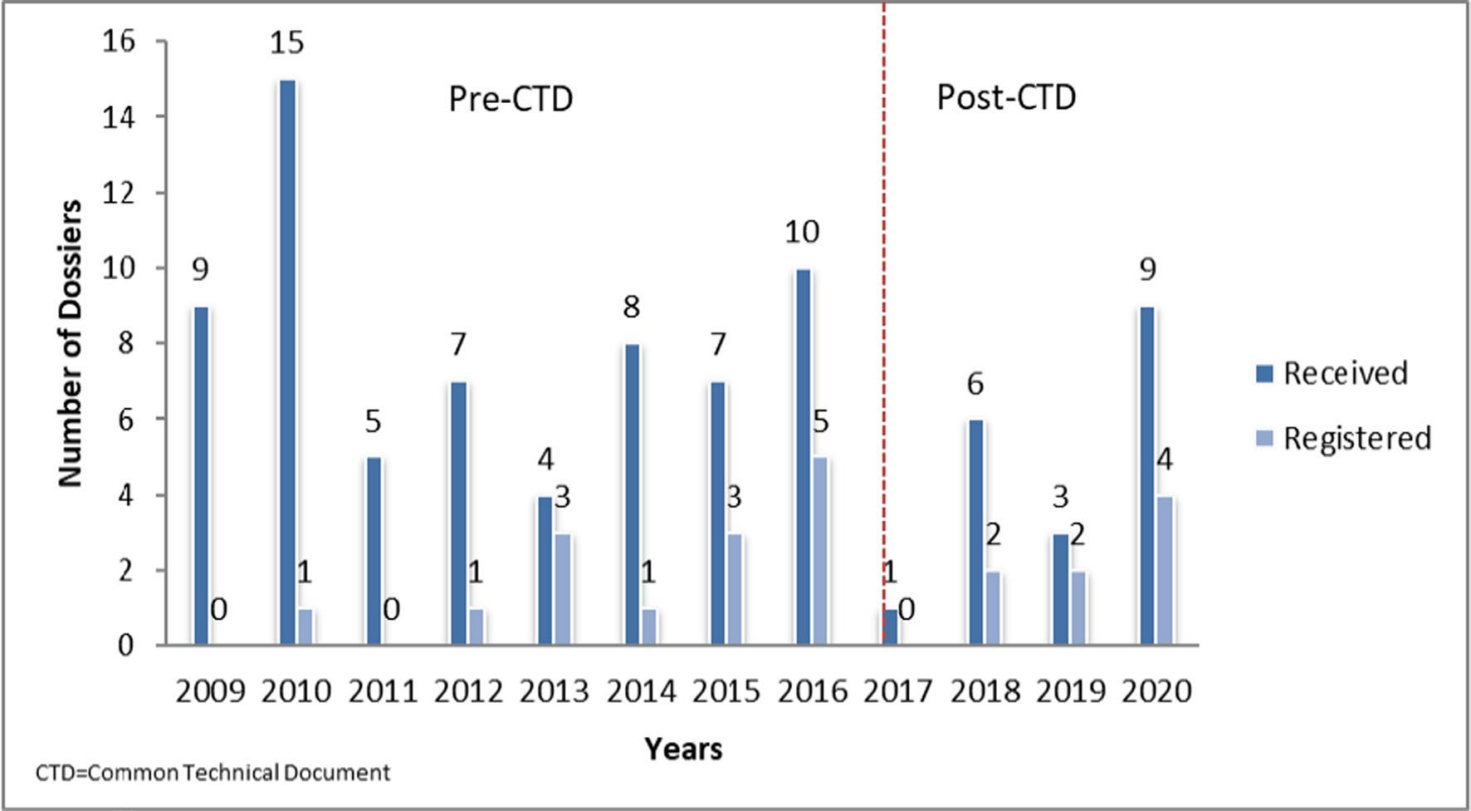
Number of herbal medicine dossiers received and registered annually by TMDA (2009–2020)

Figure 2 shows the number of herbal medicine applications that were received and registered by the TMDA each year. The online submission of herbal medicine applications in Tanzania started in 2020, and the requirements for submission in Common Technical Document (CTD) format were introduced in February 2017. There was no significant difference in the proportion of applications received and approved before the introduction of the CTD (2009–2016) and after the introduction of the CTD (2017–2020) (*p* = 1).

A total of 172 plants were incorporated in the formulation of herbal medicines submitted for MA in Tanzania. The plants frequently incorporated in various herbal medicines whose applications were submitted are summarized in Table 1.

**Table 1:** Plants frequently^a^ used in herbal products submitted for market authorization in Tanzania (2009–2020) (*N* = 84)

Botanical name	Number of products containing the plant	Botanical name	Number of products containing the plant
*Zingiber officinale*	22	*Mentha piperita*	4
*Glycyrrhiza glabra*	17	*Mentha arvensis*	4
*Piper longum*	14	*Mentha sylvestris*	4
*Adhatoda vasica*	13	*Mucuna pruriens*	4
*Terminalia bellirica*	12	*Picrorhiza kurroa*	4
*Eucalyptus globulus*	11	*Capsicum annum*	3
*Curcuma longa*	8	*Cichorium intybus*	3
*Cinnamomum camphora*	8	*Commiphora mukul*	3
*Ocimum sanctum*	8	*Embelia ribes*	3
*Piper nigrum*	8	*Emblica officinalis*	3
*Aloe barbadensis*	6	*Alpinia galanga*	3
*Terminalia chebula*	6	*Myroxylon balsamum*	3
*Boerhavia diffusa*	5	*Pinus roxburghi*	3
*Cinnamomum zeylanicum*	5	*Piper cubeba*	3
*Gaultheria procumbens*	5	*Plumbago zeylanica*	3
*Syzygium aromaticum*	5	*Syncarpia glomulifera*	3
*Withania somnifera*	5	*Solanum xanthocarpum*	3
*Asparagus racemosus*	4	*Tinospora cordifolia*	3
*Eclipta alba*	4	*Vitex negundo*	3

^a^Frequently used = plant included in 3 or more herbal products

*N* = total number of dossiers received and analysed

**Fig. 3:**
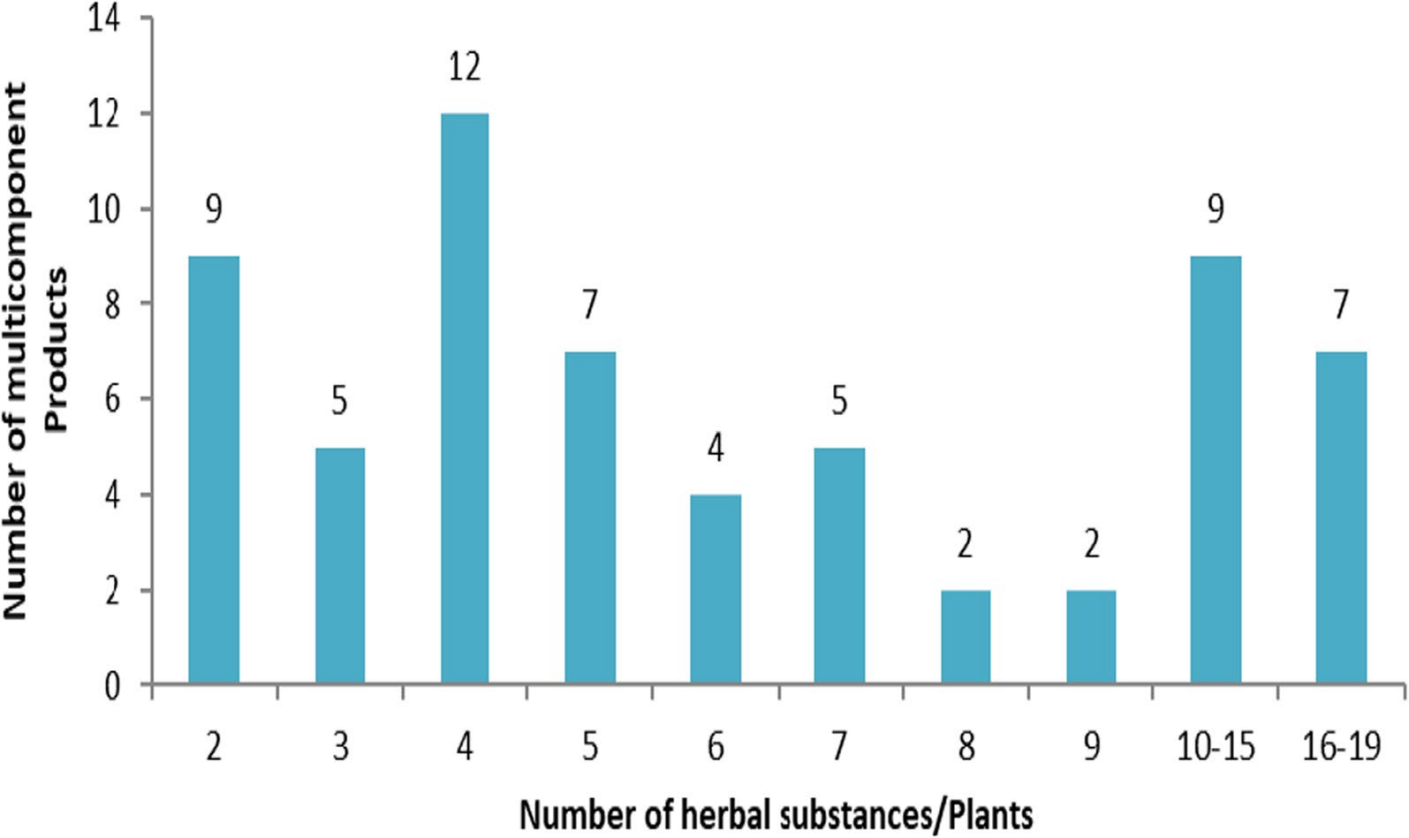
Number of multicomponent product applications with the number of herbal substances/plants in the products

As many as 75% (63/84) of the herbal product applications contained more than one plant or herbal substance, while 25% (21/84) were made with a single herbal substance or plant. The number of substances or plants used ranged between 2 and 19 per product as shown in Fig. 3. Details on the various plants included in the multicomponent herbal products that were received and granted MA are shown in Additional file 1. Commonly received plants used in multicomponent herbal products that were evaluated and were not registered in Tanzania are shown in Additional file 2.

About 74% (62/84) of the received herbal medicines were classified by the TMDA as over-the-counter medicines, 22% (19/84) prescription only, 2% (2/84) as pharmacy only and 1% (1/84) as general sales medicines. For registered herbal medicines, 59% (13/22) were over-the-counter medicines, 32% (7/22) prescription only, 4.5% (1/22) pharmacy only and 4.5% (1/22) were general sales medicines.

### Safety

#### Preclinical studies

Preclinical safety studies data were contained in 38% (32/84) of the herbal medicine dossiers that were received. Twenty-two percent of the studies were conducted on both rodents and nonrodents with the majority (78%) of the studies being conducted on rodents only. Details of the types of toxicity studies performed are presented in Table 2.

**Table 2:** Frequency of preclinical toxicity studies in herbal medicine dossiers submitted in Tanzania (2009–2020)

Toxicity study type	Number of applications (%) (*n* = 32)
Acute single dose	32 (100)
Sub-chronic/repeat dose	9 (28)
Chronic	11 (34)
Developmental	3 (9)
Carcinogenicity	6 (19)

*n* = total number of dossiers with preclinical studies

#### Clinical studies

About 32% of herbal medicine dossier applications contained clinical safety studies. Most of the submitted safety data 70% (19/27) were from literature sources, while 30% (8/27) were from studies conducted by the manufacturers themselves.

Regarding the manufacturers’ studies, about 75% (6/8) of their products were reported to have no ARs or side effects while only 25% (2/8) of the products were declared to have ARs. The declared ARs included hypernatremia (1/8) and alkalosis (1/8).

The majority (95.2%) of the herbal medicines were reported by the applicants to have no interactions with other medicines, while only a few (4.8%) were reported to interact with other medicines. Overdose data were mostly not provided except in few dossiers (2.4%).

### Safety data submitted for commonly used plants in herbal product applications

A description of the safety data submitted for ten plants commonly used in herbal product applications submitted in Tanzania is shown in Table 3.

**Table 3:** Safety data submitted for ten frequently used plants in the herbal products applications (2009–2020)

Botanical name	Total number of products	Preclinical safety	Clinical safety
		Number of products (%)	Number of products (%)
*Zingiber officinale*	22	11 (50)	6 (27)
*Glycyrrhiza glabra*	17	6 (35)	7 (41)
*Adhatoda vasica*	13	9 (69)	6 (46)
*Eucalyptus globulus*	11	1 (9)	3 (27)
*Piper longum*	10	9 (90)	5 (50)
*Terminalia bellirica*	9	7 (78)	3 (33)
*Curcuma longa*	8	5 (63)	2 (25)
*Cinnamomum camphora*	8	0 (0)	2 (25)
*Ocimum sanctum*	8	3 (38)	4 (50)
*Piper nigrum*	8	4 (50)	2 (25)

Some of the plants used in the herbal products submitted for MA have documented toxicity risks or adverse reactions (ARs) in the literature. Therefore, we searched the applications to see if there were any references from the literature or documentation on the safety of the ten plants used in the applications. The results of this analysis are presented in Table 4.

**Table 4:** Safety data submitted in herbal products applications with herbal components with documented risks of adverse reactions or toxicity (2009–2020)

Botanical name	Total number of products	Preclinical safety	Clinical safety	References
		Number of products (%)	Number of products (%)	
*Echinacea spp. (E. angustifolia and E. purpurea)*	2	0 (0)	0 (0)	[41]
*Gingko biloba*	2	1 (50)	1 (50)	[42]
*Panax ginseng*	2	1 (50)	1 (50)	[42, 43]
*Valeriana officinalis*	2	0 (0)	2 (100)	[44, 45]
*Atropa belladonna*	1	0 (0)	1 (100)	[46, 47]
*Serenoa repens*	1	1 (100)	1 (100)	[48, 49]
*Myristica fragrans*	1	0 (0)	1 (100)	[50, 51]
*Solanum nigrum*	1	1 (50)	0 (0)	[52]

### Quality parameters with safety implications

The herbal medicine application dossiers contained data on both the herbal substances/ingredients and the finished herbal products. Specifications for the herbal substances/ingredients were provided in 82% (69/84) of the products. The test parameters for the active herbal substances/ingredients used in different monographs either singularly or in combinations which included In-house monographs [58], Indian [6], European [6], British [3], The Committee on Herbal Medicinal Products (HMPC) [3], WHO [2], The European Scientific Cooperative on Phytotherapy (ESCOP) [2] Chinese [1], Ayurvedic [1], US Pharmacopeia (USP) [1] and the Association of Official Analytical Collaboration (AOAC) [1]. Only 30% (25/84) of the submitted herbal dossiers indicated the methods of preparation of the herbal substances/ingredients.

Of the submitted dossiers, 82% (69/84) contained herbal substance specifications, of which 52% (36/69) contained specifications for purity and contaminant tests. The tests conducted are shown in Table 5. Regarding the finished herbal products (93%) 78/84 contained finished product specifications of which 60 (77%) 60/78 contained specifications for purity and contaminant tests. The results are given in Table 6. Stability studies to establish shelf-life for finished herbal products were conducted on 80% (67/84) of the products.

**Table 5:** Quality parameters with safety implications tested in herbal substances submitted for marketing authorization (2009–2020)

Tests parameters	Number of products (%) (*n* = 69)
Mycotoxins	6 (9)
Pesticides/fumigants	8 (12)
Total microbial count	30 (43)
Total fungal count	15 (22)
Yeast only count	14 (20)
Mould counts	27 (39)
Pathogens (*S. aureus, E. coli, Salmonella* spp, *P. aeruginosa*)	26 (38)
Heavy metals (arsenic, mercury, lead and cadmium)	24 (35)

*n* = total number of dossiers with herbal substances specifications

**Table 6:** Quality parameters with safety implications tested in finished herbal medicines submitted for marketing authorization (2009–2020)

Tests parameters	Number of products (%) (*n* = 78)
Total microbial count	15 (19)
Total fungal count	3 (4)
Yeast only count	11 (14)
Mould counts	14 (18)
Pathogens *(S. aureus, E. coli, Salmonella spp, P. aeruginosa)*	18 (23)
Heavy metals (arsenic, mercury, lead and cadmium)	11 (14)

*n* = total number of dossiers with finished herbal medicines Specifications

### Quality assurance

Evidence on cultivation and collection of the plant materials by considering GACP was submitted in only a few (4%) products. Out of the submitted preclinical studies, 13% (5/38) declared conducting the studies following GLP. For clinical studies submitted 23% (9/39) demonstrated compliance with GCP. Evidence of being inspected by the regulatory authorities in the country of origin to verify compliance with GMP was submitted for 42% (35/84) of the products. Of note is that TMDA also conducts actual verification of GMP compliance regardless of the submission of the GMP certificate from the regulatory authority of the country of manufacture as per the requirements [53]. All 22 herbal medicines that were granted MA were therefore verified for GMP compliance by the TMDA.

### Safety data described in the patient information leaflets/package inserts

The frequency of safety information declared on patient information leaflets/package inserts or a summary of product characteristics is shown in Table 7.

**Table 7:** Safety data declared in package inserts of herbal medicines submitted for marketing authorization (2009–2020)

Safety category	Number of products (%) *N* = 84
Contraindications	42 (50)
Warnings or precautions	27 (32)
Side effects	25 (30)
Overdose	16 (19)
Pregnancy and lactation (not recommended)	13 (16)
Interactions	5 (6)
Pregnancy and lactation (conditional recommendation)	3 (4)

*N* =total number of dossiers received and analysed

The commonly declared side effects of herbal medicines in the package inserts, patient information leaflets or summary of product characteristics submitted for review were nausea [10], abdominal discomfort [8], headache [7], hyperacidity [6], diarrhoea [5], vomiting [5], gastrointestinal complaints [3], skin rashes [3] and contact dermatitis [2]. The side effects declared for the herbal products granted MA are shown in Table 8.

**Table 8:** Side effects declared in the registered herbal medicines package inserts or summary of product characteristics (2009–2020)

Plants contained in the herbal product	Possible side effects declared in the product (*n* = 22)
*Syncarpia glomulifera, Eucalyptus globulus and Myristica fragrans, Camphor*	Hypersensitivity reactions
*Euphorbia prostrata*	Dry mouth, nausea, gastralgia, abdominal pain, dyspepsia, dizziness, headache, skin and subcutaneous tissue disorders, contact dermatitis, immune system disorders, hypersensitivity
*Glycyrrhiza glabra, Zingiber officinale, Emblica officinalis*	Flatulence, laxative effect
*Hedera helix*	Nausea, vomiting, laxative effect
*Serenoa repens*	Dizziness, headache, nausea, vomiting, constipation, and diarrhoea. liver or pancreas problems
*Vaccinium myrtillus, Betacarotene*	Gastrointestinal distress, skin rashes, drowsiness

*n* = number of package inserts/summary of product characteristics with declared side effects

## Discussion

The analysis of the herbal products data with a focus on safety submitted to TMDA from 2009 to 2020 showed that the majority of the applicants did not provide evidence to support preclinical and clinical safety. It was observed that most of the herbal medicines submitted for MA were multicomponent herbal products. Most of the applicants did not provide evidence on quality parameters with safety implications.

### Features of herbal medicines submitted for marketing authorization

The analysis of the dossiers submitted in this study showed that there was no noticeable difference in the trend of the number of applications received and granted MA from 2009 to 2020 after the introduction of the CTD format in 2017. This shows that introduction of CTD did not affect the rate of herbal dossier submission to TMDA.

In this study, only a few herbal medicines (26%) were granted MA based on complete submissions of evidence for the assessors to determine whether the product is safe to be consumed by the populations. Similar results of low registration of traditional herbal medicine applications were reported by some of the European regulatory authorities in Denmark (11%), France (19%), Romania (24%) and Cyprus (25%) according to the survey report of the European Medicines Agency (EMA) of December 2017 [54]. This low registration rate in Tanzania could be explained by the lack of scientific information to substantiate registration of the products or a lack of knowledge for some manufacturers of herbal products on proper compilation of the dossiers and TMDA requirements, as many queries for submission of additional information were requested.

Assessment of the products submitted showed that 172 plant species were used for the formulation of herbal medicines with the ten most commonly used plants being *Z. officinale, G. glabra, A. vasica, E. globulus, P. longum, T. bellirica, C. longa, C. camphora, O. sanctum* and *P. nigrum.* The same plants were found to be commonly registered in Ghana by the Ghana Food and Drugs Authority [55] and in Nigeria by the National Agency for Food and Drug Administration and Control [56].

### Safety

#### Combination of herbal medicines

A higher proportion (68%) of herbal medicines granted MA were formulations that contained several plants or active herbal ingredients. The multicomponent nature of these products is the same as those produced in China and other Asian countries, where manufacturers of herbal medicines are adopting the theory of Asian and Chinese traditional medicine practices whereby complex formulations are used to attain synergism or complementary effects [57]. The situation is different in European Union countries where a study conducted by Wieland Peschel in 2014 showed that the multicomponent herbal products that were granted MA were only 34% for traditional herbal medicinal products and 19% for well-established use herbal medicines [37]. The use of multiple plants in one formulation however must be justified by manufacturers and there are very few studies showing the beneficial effects of complex multiple herbal products in curing diseases [58–61].

Rigorous scrutiny of multicomponent product dossiers is recommended due to their potential to cause herb–herb interactions that may cause adverse events [62]. This potential for herb–herb interactions has been demonstrated in previous studies, for instance, liquorice (*G. glabra*) root interacted with either *Veratrum nigrum, Sargassum pallidum* or *Euphorbia pekinensis* [19]. In this study, the results showed that *G. glabra* was combined with multiple different plants in 17 preparations, nonetheless, preclinical safety was submitted in only 35% of the applications and clinical safety in only 41%. Manufacturers have mixed several different plants in their preparations of which most of the combinations have not been studied for their safety as evidence was not available in the submitted dossiers.

The results of this study also showed that piper species (*P. longum, P. nigrum and P. cubeca*) were combined with *Adhatoda vasica* in nine preparations together with other herbals. Additionally, Piper species were combined with *Curcuma longa* (turmeric) in four preparations. Piper species have been documented to interact with herbal and conventional medicines such as *R. rosea*, *C. longa*, *A. vasica,* nevirapine, phenytoin, propranolol, theophylline and verapamil by inhibiting cytochrome P450 or isoenzymes or P-glycoprotein, resulting in an increase or decrease in their bioavailability [63, 64]. However, those studies were limited to in vitro and animal models with very little clinical evidence in humans, which was the same as observed in this study, whereby preclinical safety data were submitted for 90% of the *P. longum* and 50% of the *P. nigrum* products. Clinical safety data were submitted in 50% *P. longum* and 25% *P. nigrum* products. It was also observed that these documented interactions in the literature were not included in the package inserts to warn consumers.

#### Safety data

The TMDA guidelines for the submission of documentation for MA of herbal medicinal products clearly state that data to support evidence of safety should be submitted either as reports or from the literature [34]*.* However, only a few preclinical (38%) and clinical (32%) safety data were submitted in herbal product applications. This lack of evidence from the applicants could be associated with the fact that traditional herbal medicines used for a long time are presumed to be safe and could warrant waivers, as done by the regulatory authorities in some countries. The WHO recommends that there should not be stringent regulatory requirements on safety data if a product has been traditionally used for a long time and is proven to be harmless, however, evidence of safety should be provided through literature sources and references [6].

In this study, it was found that applications for Echinacea species (*E. angustifolia* and *E. purpurea*) products lacked preclinical and clinical safety data despite the availability of safety data in the literature conducted over the years since the 1950s, as well as case studies and spontaneous reports from the regulatory authorities [41]. In this case, the inclusion of literature data to support safety would have prevented unnecessary delays and resubmissions to the TMDA.

A similar observation was made for products containing eucalyptus oil, where most of the dossiers lacked preclinical and clinical safety data despite literature documentation on its toxicity, AEs with prolonged use and in children [65, 66]. Inclusion of the safety data from the literature and a precautionary warning to “avoid prolonged use and not to give to children under 2 years of age” might have been sufficient evidence to support the product’s safety for MA.

Other herbal medicines with documented adverse effects and/or toxicity in the literature, such as *P. ginseng*, *G. biloba* and *S. nigrum* [42, 52], were used in the formulations in the submissions and should have been accompanied by documentation to support the safety of the products. However, only half of the products for *P. ginseng* and *G. biloba* had submitted preclinical and clinical safety data, and *S. nigrum* had no clinical safety data even though there were some cases of toxicity in the literature [52].

In this study, products containing *V. officinalis, A. belladonna* and *M. fragrans* were granted MA based on the submission of clinical safety and efficacy data, despite the lack of submission of preclinical data according to TMDA and WHO guidelines [6, 34]. This shows the importance of systematic clinical trials to make evidence-based decisions on the benefit–risk profile of a herbal product [7]. Although manufacturers are expected to understand this importance, the results showed that the majority (70%) of them did not conduct clinical studies of their products and relied only on the literature data. The same was observed in a study conducted in the Netherlands in 2001, whereby most of the evidence from the applications was sourced from the literature [36].

Toxicity data were only submitted for 38% of products as part of preclinical safety studies, which were mainly on acute toxicity, followed by chronic, repeat dose/ sub-chronic, carcinogenicity and developmental toxicity. This low submission rate of toxicity data was also observed in a previous study in the Netherlands [36]. Submission of toxicity studies is important since carcinogenicity, developmental and reproductive toxicities cannot be determined by long-term use alone without conducting studies [67]. *Gingko* was used for many years however, it was found to have carcinogenicity potential in preclinical studies in vitro and in vivo studies done in rodents [68, 69]. *Ephedra* products have also been used for a long time and it was discovered that they can cause cardiovascular toxicity [70, 71]*.*

Despite the WHO recommendation on granting waivers for submission of toxicity studies for long traditional used herbal products, they also recommend that for herbal products that have toxicity, documented risk assessment information should be provided together with documentation on long-term use safety otherwise toxicity studies should be performed [6]. The applicants did not follow the recommendation since they did not provide any documentation from the literature to support long-term use safety or the absence of toxicity risks.

As with conventional medicines, documented evidence of developmental and reproductive toxicity may be required if the herbal product is intended to be used by women with childbearing potential, pregnant, or lactating. [34]. In this study, developmental and reproductive toxicity studies were not submitted in the majority (96%) of the applications. This can be explained by the fact that these studies are usually not necessary for products with documentation in the literature on long-term traditional use unless there is some safety concern or the product changes fertility, hormones or has an effect on the endocrine system [72].

Herbal medicines such as *C. camphora* essential oils, when used in high doses have been observed to cause maternal toxicity in pregnant animal studies and in clinical studies which emphasizes the necessity of conducting reproductive toxicity studies [73]. In contrast, in this study, there were no preclinical data submitted for *C. camphora* with few (25%) clinical safety data. Furthermore, package inserts did not include any warnings or precautions for consumers in the regarding the potential toxicity.

#### Quality data with safety implications

The results showed that tests on contaminants such as heavy metals, pesticides, fumigants, mycotoxins, microbial counts, fungal and mould were submitted in only half (52%) of the active herbal substances and in 77% of the finished herbal product specifications. This is contrary to the TMDA guidelines which require specifications for both active herbal substances and finished herbal products to include tests to verify purity [34]. The absence of such important tests explains the low rate of approvals in this study since contaminants have a direct influence on the outcomes of the safety assessment of a herbal product [74].

Heavy metal contamination in herbal medicines may originate from the environment, manufacturing processes, or may be intentionally added by manufacturers in belief of their medicinal potential [75]. Tests for the control of heavy metals are critical for the authorization of a herbal product since even very small amounts can be very toxic [76]. In this study, tests for heavy metals (mercury, lead, cadmium and arsenic) were included in only 35% and 14% of the active substances and finished herbal product specifications, respectively. A study conducted in Malaysia by the Drug Regulatory Authority found that 22% of *Eugenia dyeriana* preparations on the market were contaminated with lead [77]. It is therefore important to monitor heavy metals both during MA assessments and post-market to ensure continuous compliance.

Mycotoxins such as aflatoxins are by-products of fungal contamination in plants and are a human health hazard with fatal outcomes in some cases [78]. Contamination can occur during post-harvesting processes, storage or transportation [79]. The results of this study showed that the majority of the submissions (91%) did not include mycotoxin tests in the raw material specifications despite being among the regulatory requirements for MA of herbal products [34, 80]. Furthermore, evidence of possible contamination has been reported in previous studies of medicinal herbs in Spain where over 96% were found to be contaminated with aflatoxins and other mycotoxins [81] and aflatoxin contamination in 43% of crude herbs and 64% of finished herbal products in India [82].

Agrochemical contaminants such as insecticides, herbicides and fungicides should be controlled in herbal medicine raw materials due to possible contamination from soil, farming/cultivation, water sources and post-harvest processing [79]. The absence of these contaminants therefore indicates the manufacturer’s compliance with GACP and GMP practices during all stages, from cultivation to production and storage. However, in this study, few (12%) manufacturers included tests for pesticides or fumigant residues in the herbal substance specifications. This could imply that manufacturers did not follow GACP and GMP standards as only a few applicants (4%) provided evidence of GACP for the raw materials and 42% for GMP compliance. A study conducted in the USA on herbal medicines sourced from China showed that 36% of the samples were contaminated with pesticide residues [83] and a study on five ginseng plants in China found high levels of four types of organochlorinated pesticide residues [84]. The importance of verification tests on acceptable levels of these contaminants by the manufacturers cannot be emphasized enough.

Few submissions in this study included test results for total microbial, fungal, mould, and yeast counts of active herbal substances and finished herbal products. These tests are critical in ensuring consumer safety [79]. A study in Brazil showed that more than 50% of the herbal medicines that circulate on the market were contaminated with microorganisms [85], while another study identified 42 fungal species contaminating raw materials of *T. cordifolia* and *M. fragrans* [86]. Based on the observations made, it is evident that these critical tests could not be waived by the regulatory assessors.

#### Safety data in patient information leaflets or summary of product characteristics

Most of the key safety data were not included in the patient information leaflets, with little information on warnings, precautions, side effects, overdose, pregnancy and lactation, or interactions. For example, *G. glabra* (liquorice) has been documented to cause ARs in the cardiovascular system including cardiac arrhythmias, hypertension, hypokalemia myopathy and hypermineralcorticoidism and is therefore not recommended for use for a long period of time [87]. However, the applicants did not include these reactions in the package insert for consumers and healthcare workers to be cautious except for one product which mentioned “the adverse events were uncommon, and the consumers should avoid prolonged use” without providing reasons. This pattern was also observed for *C. camphora* which has been documented to cause hepatotoxicity but was not mentioned in the package inserts [88]. Asian ginseng has been documented to interact with digoxin [89], however no documentation of any interaction was provided in the package inserts or in the summary of product characteristics. This lack of information could be explained by the fact that most of the toxicity or adverse reactions were not supported by evidence from sufficiently large systematic RCTs.

A similar lack of safety information was observed in a United Kingdom study, whereby 75% of the five commonly used herbal products on the market did not contain key information on safety [90]. Patient information is important to ensure that consumers understand important safety information such as possible ARs, side effects, contraindications, warnings and precautions to avoid unexpected outcomes from the medicines [91]. A survey conducted among consumers of herbal medicines in the UK showed that most of the herbal products’ consumers had little knowledge about possible safety issues of the products they consumed, and 40% presumed herbal medicines to be safe [92]. This lack of awareness of possible ARs might lead to few reports of adverse reactions due to herbal medicines being reported to the regulatory authorities.

### Strengths and limitations

A major strength of this study is the access to the dossiers and the TMDA database for analysis of the information submitted over a wide span of 12 years. A major limitation of this study is the possibility of bias, as with any document analysis, one must work with the data that are available.

### Recommendations

Manufacturers should build capacity on knowledge of herbal medicine regulations and guidelines for MA in various countries to address the serious problem of data deficiencies. Regulatory authorities should conduct a series of sessions with manufacturers and marketing authorization holders that include support, training, and informing them about the requirements in different sections of the guidelines to reduce future resubmissions and delays in the application process. This will also help to avoid the circulation of the same products in the market as food supplements since once the products are queried or refused due to a lack of adequate data, there is a high probability that they end up in the market with new claims as food supplements. There is also a need for manufacturers and stakeholders to invest more in research and development for herbal medicines and generate safety and efficacy data to support market authorization.

A detailed future study could be carried out on the comments issued to the applicants and their responses to identify frequently occurring deficiencies issued in each section and the reasons for not providing the data to inform all stakeholders.

## Conclusions

Analysis of the herbal medicine dossiers submitted to TMDA for application for marketing authorization from 2009 to 2020 showed major deficiencies in safety data to establish the safety profile of herbal medicines to support MA. Many of the products contained various herbal ingredients in one product without evidence from the literature to justify the safety of the combinations and absence of interactions. Very few products contained safety information for consumers and healthcare workers. The lack of scientific information and evidence from systematically conducted RCTs makes it difficult to make regulatory decisions to grant MA for herbal products. Manufacturers need to provide evidence to support the safety of their products for evidence-based regulatory decisions and to avoid multiple reviews of the applications.

We believe that the results of this study will assist in the identifying gaps in the compilation and review of safety data for MA submissions of herbal medicines. This will inform manufacturers, researchers, and regulators on areas for improvement in data collection, organization, benefit–risk assessment, and decision-making. It will also assist researchers and sponsors of herbal medicines in identifying areas with gaps for research investments.

### Supplementary Information

**Additional file 1.**Plants combined in multicomponent herbal products registered in Tanzania (2009-2020)

**Additional file 2.**Commonly received plants used in multicomponent herbal products that were evaluated and were not registered in Tanzania (2009-2020)

## Data Availability

The data are not available due to privacy and confidentiality.

## Abbreviations

AOAC: Association of Official Analytical Collaboration
AR: Adverse reaction
CTD: Common technical document
EMA: European Medicines Agency
ESCOP: European Scientific Cooperative on Phytotherapy
GACP: Good Agricultural and Collection Practice
GCP: Good clinical practice
GLP: Good laboratory practice
GMP: Good manufacturing practice
HMPC: The Committee on Herbal Medicinal Products
ICH: International Council for Harmonization of Technical Requirements for Pharmaceuticals for Human Use
MA: Marketing Authorization
RCT: Randomized Controlled Clinical trial
TMDA: Tanzania Medicines and Medical Devices Authority
USP: US Pharmacopeia
WHO: World Health Organization
