# Supplementary material for: Safety profile of herbal medicines submitted for marketing authorization in Tanzania: a cross-sectional retrospective study
Source: J Pharm Policy Pract. 2023 Nov 20;16:149. doi: 10.1186/s40545-023-00661-x (PMC10658996; doi:10.1186/s40545-023-00661-x)
Supplement: Supplementary file 1 — Additional file 1.Plants combined in multicomponent herbal products registered in Tanzania (2009-2020) [file 40545_2023_661_MOESM1_ESM.docx]

| **Additional File 1: Plants combined in multicomponent herbal products registered in Tanzania (2009-2020)**  **Table 1: Plants combined in multicomponent herbal products registered in Tanzania (2009-2020)** | |
| --- | --- |
| **No.* of plants** | **Botanical Names** |
| 2 | *Mentha sylvestris, Mentha piperata*, Menthol |
| 3 | *Glycyrrhiza glabra, Zingiber officinale, Emblica officinale and Levomenthol* |
| 4 | *Mentha piperata, Cinnamomum camphora, Gautltheria fragrantissima, Eucalyptus globulus* |
| 4 | *Camphor, Syncarpia glomulifera , Menthol, Eucalyptus globulus , Myristica fragrans , Thymus vulgaris* |
| 4 | *Tribulus terrestris, Boerhavia diffusa, Bauhinia variegate, Crataeva nurvala* |
| 5 | *Syzygium aromaticum,Eucalyptus globulus, Cinnamomum camphora, Cinnamomum zeylanicum and Syncarpia glomulifera* Menthol |
| 6 | *Mentha viridis, Cinnamomum camphora Phyloganthus curviforus, Cinnamomum tamala, Eucalyptus Globulus Glychirrhiza glabra* |
| 7 | *Adhatoda vasica ,Ocimum sanctum,Abbies webbiana Glycyrrhiza glabra ,Curcuma longa ,Piper longum, Inula racemosa* |
| 7 | *Withania somnifera, Glycyrrhiza glabra, Zingiber officinale, Piper longum, Embelica officinalis, Terminalia chebula,Terminalia bellirica* |
| 9 | *Adhatoda vasica, Ocimum santum, Piper longum, Zingiber officinale, Glycyrrhiza glabra, Embelica officinalis , Terminalia chebula, Terminalia cordifolia & Tinospora cordifolia* |
| 10 | *Ocimum sanctum, Glycyrrhiza glabra, Curcuma longa, Zingiber officinale, Adhatoda vasica, Solanum indicum, Inula racemosa, Piper cubeba, Terminalia bellirica, Aloe babadensis & Menthol* |
| 11 | *Glycyrrhiza glabra, Zingiber officinal, Ocimum santum , Curcuma longa , Adhatoda vasica , Solanum indicum , Inula racemosa , Piper cubeba , Terminalia bellirica , Aloe barbadensis* and Levo Menthol |
| 16 | *Adhatoda vasica, Glycyrrhiza glabra, Ocimum Sanctum, Alpinia galanga, Cinnamomum zeylanicum, Terminalia bellirica, Abies webbiana Curcuma longa, Pistacia integerrima, Solanum xanthocarpum, Syzygium aromaticum, Mentha piperita , Cinnamomum camphora, Piper nigrum, Piper longum, Zingiber officinale* |
