# Supplementary material for: Safety profile of herbal medicines submitted for marketing authorization in Tanzania: a cross-sectional retrospective study
Source: J Pharm Policy Pract. 2023 Nov 20;16:149. doi: 10.1186/s40545-023-00661-x (PMC10658996; doi:10.1186/s40545-023-00661-x)
Supplement: Supplementary file 2 — Additional file 2.Commonly received plants used in multicomponent herbal products that were evaluated and were not registered in Tanzania (2009-2020) [file 40545_2023_661_MOESM2_ESM.docx]

| **Additional File 2: Commonly received plants used in multicomponent herbal products that were evaluated and were not registered in Tanzania (2009-2020)**  **Table 2: Commonly received plants used in multicomponent herbal products that were evaluated and were not registered in Tanzania (2009-2020)** | |
| --- | --- |
| **Number of plants** | **Botanical Names** |
| 2 | *Cichorium intybus, Erycoma longifolia* |
| 4 | *Mentha piperata, Salvia rosmanus, Eucalyptus globulus, Gautheria procumbens* |
| 4 | *Mentha sylvestris, Glycyrrhiza glabra, Zingiber officinale, Emblica officinalis,* |
| 4 | *Adhatoda vasica, Glycerriza glabra, Piper longum, Alpinia galanga* |
| 4 | *Holarrhena dysentrica, Berberis aristata ,Embellica officinalis, Terminalia chebula* |
| 5 | *Glycyrrhiza glabra, Terminalia bellirica, Zingiber officinale, Curcuma longa, Mentha piperata* |
| 5 | *Capsicum frutescens, Myroxylon balsamum ,Myroxylon balsanum, Glycyrrhiza glabra, Pimpinella anisum* |
| 5 | *Plectranthus amboinicus , Zingiber officinale , Mentha piperata , Cinnamomum zeylanicum , Eucalyptus globulus* |
| 5 | *Anethum graveolens, Caram carvi, Cinnamomum zeylanicum, Syzygium aromaticum, Elletaria cardamomum* |
| 5 | *Cortex phellodendri, Cortex cinnamomi, Flos caryophylli, Pericarpium citri, Menthol, Glycyrrhiza glabra* |
| 5 | *Cedrus deodara, Cinnamomum camphora, Saccharum officinarum, Mentha piperata, Capsicum annum* |
| 6 | *Capsicum annum, Boswelia serrata, Boerhavia diffusa, Vitex negundo, Eucalyptus globulus, Gaultheria procumbens* |
| 6 | *Tribulus terrestris, Withania somnifera, Mucuna pruriens, Orchis latipolia, Chlorophytum arundinaceum , Ricinus communis* |
| 8 | *Withania somnifera, Saraca indica, Asparagus racemous, Vitits venifera, Cuminum cyminum, Tinospora cordifolia, Symplocos racemosa, Nardostachys jatamansi* |
| 10 | *Comiphora mukul, Paederia foetida, Pluchea lanceolata, Zingiber officinale, Vitex negundo, Ricinus communis, Lepidium sativum, Colchicum leteum, Smilax glabra, Strychos nuxvomica* |
| 11 | *Adhatoda vasica, Solanum xanthocarpum, Ocimum santum, Glycyrrhiza glabra, Zingiber officinale, Piper longum, Mentha spicata, Pudica satva, Hedychium spicatum,Viola odorata, Abies webbiana* |
| 16 | *Terminalia chebula, Terminalia bellirica, Embica officinalis, Melia azadirachta , Vitex negundo, Cissus quadrangularis, Commiphora mucul , Costus speciosus, Gossypium hirsutum, Pinus longifora, Cinammomum camphora, Apium graveolens, Cedrus deodara, Syncarpia glomulifera , Eucalyptus globulus, Melaceca leucadendron* |
| 16 | *Asparagus racemosus, Sida cordifolia, Tribulus terrestris, Mucuna pruriens, Withania somnifera, Tinospora cordifolia, Emblica officinalis, Chlorophytum arundinaceum, Argyreia speciosa,Zingiber officinale, Piper nigrum, Piper longum, Tacca aspera, Smilax china, Ipomoea digitata, Boerhavia diffusa* |
| *19* | *Saraca indica, Symplocos racemosa, Bryonia laciniosa, Terminalia chebula, Adhatoda vasica, Ficus bengalensis, Cedrus deodara, Hygrophila spinosa, Asparagus racemosus, Emblica officinalis, Andropogon muricatus, Nelumbium speciousum, Cuminum cyminum, Zingiber officinale, Mesua ferrea, Caryophyllus aromaticus, Piper cubeba, Nardostachys jatamansi, Bombax malabaricum* |
